# Supplementary material for: Particulate reshapes surface jet dynamics induced by a cavitation bubble
Source: Nat Commun. 2025 Aug 14;16:7562. doi: 10.1038/s41467-025-62936-y (PMC12354815; doi:10.1038/s41467-025-62936-y)
Supplement: Supplementary file 1 — Supplementary Information [file 41467_2025_62936_MOESM1_ESM.pdf]

Supplementary Information

Particulate Reshapes Surface Jet Dynamics Induced by a Cavitation Bubble

Xianggang Cheng[1], Xiao-Peng Chen[1,2*], Zhi-Ming Yuan[3], Laibing Jia[3*]

[1]School of Marine Science and Technology, Northwestern Polytechnical University, Xi'an, 710072, China.
[2]Research & Development Institute of Northwestern Polytechnical University in Shenzhen, Shenzhen, 518057, China.
[3]Department of Naval Architecture, Ocean & Marine Engineering, University of Strathclyde, Glasgow, G4 0LZ, UK.

*Corresponding author(s). E-mail(s): xchen76@nwpu.edu.cn; l.jia@strath.ac.uk;

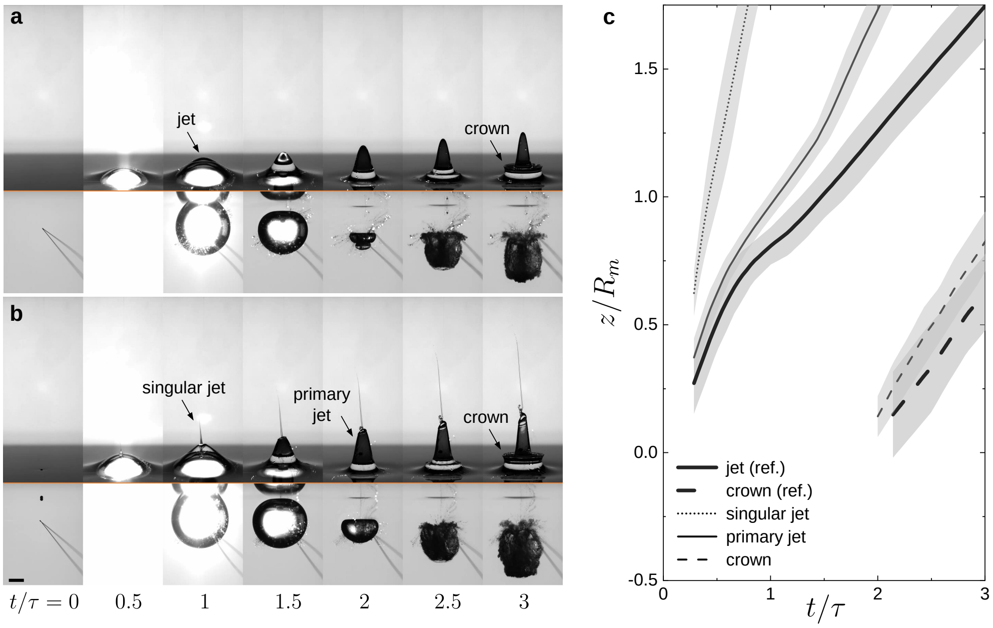

Supplementary Figure 1: **Kinematics of Mode I. a**, Reference case illustrating jet development from a flat water surface at a dimensionless depth of $\hat{h} = 0.85$. The orange line separates the views above and below the waterline. The time τ denotes the interval between the spark bubble's initiation and its maximum radius R_m. **b**, Snapshots of the Tiered Jet Mode, where a sphere initially rests on the water surface ($\hat{h} = 0.81$, $r = 500$ μm, $\rho = 4.4$, $\theta = 80.8°$). Scale bar: 5 mm. **c**, Temporal evolution of featured geometries during jet formation in the case shown in **b**. For reference, the trajectories of the jet tip and the crown top from the flat surface case at the same $\hat{h}$, corresponding to **a**, are also included. Vertical positions z are normalised by R_m, and time t is normalised by τ. The lines represent the average results from three experimental runs under the same conditions, with shadows around the lines indicating experimental uncertainty ($\hat{h} = 0.82 \pm 0.01$). Source data are provided as a Source Data file.

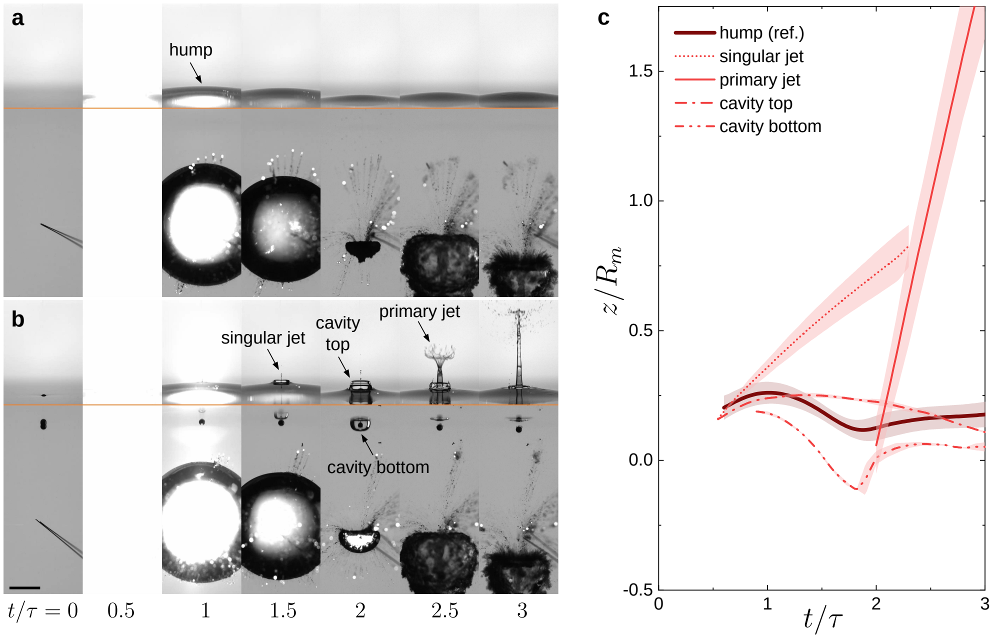

Supplementary Figure 2: **Kinematics of Mode II.** **a**, Reference case ($\hat{h} = 1.57$). **b**, Snapshots of the Jet Cavity Mode ($\hat{h} = 1.62$, $r = 500$ μm, $\rho = 4.4$, $\theta = 80.8°$). Scale bar: 5 mm. **c**, Temporal evolution of featured geometries corresponding to the case shown in **b** and the reference case shown in **a**. The lines are the average results from three experimental runs with $\hat{h} = 1.57 \pm 0.05$. Source data are provided as a Source Data file.

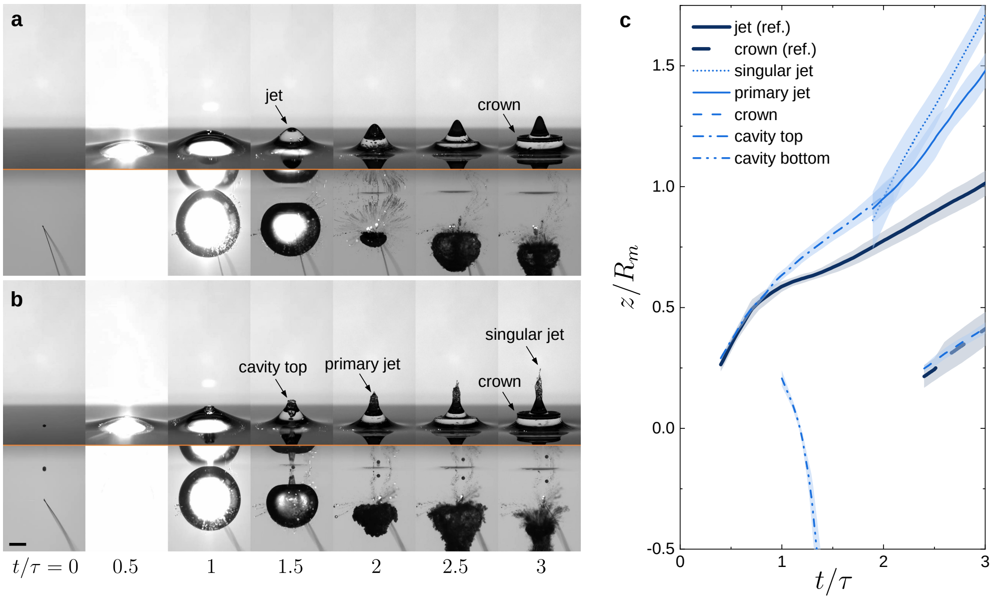

Supplementary Figure 3: **Kinematics of Mode III.** **a**, Reference case ($\hat{h} = 1.02$). **b**, Snapshots of the Cavity Venting Mode ($\hat{h} = 1.00$, $r = 500\ \mu$m, $\rho = 1.4$, $\theta = 82.1°$). Scale bar: 5 mm. **c**, Temporal evolution of featured geometries corresponding to the case shown in **b** and the reference case shown in **a**. The lines are the average results from three experimental runs with $\hat{h} = 1.03 \pm 0.04$. Source data are provided as a Source Data file.

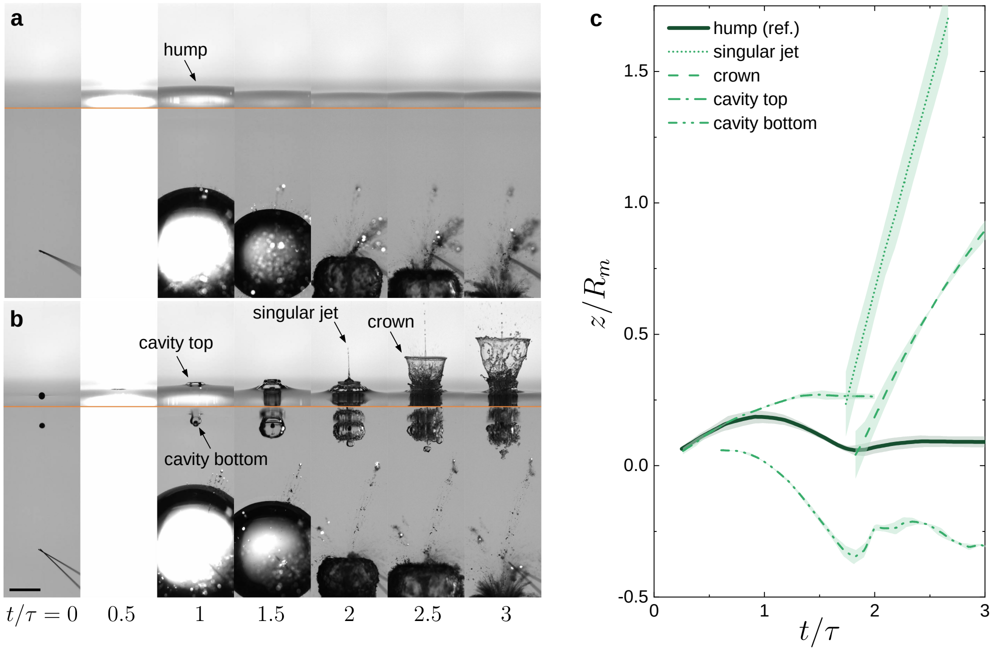

Supplementary Figure 4: **Kinematics of Mode IV. a**, Reference case ($\hat{h} = 1.95$). **b**, Snapshots of the Sealed Cavity Mode ($\hat{h} = 1.93$, $r = 500$ μm, $\rho = 4.4$, $\theta = 111.2°$). Scale bar: 5 mm. **c**, Temporal evolution of featured geometries corresponding to the case shown in **b** and the reference case shown in **a**. The lines are the average results from three experimental runs with $\hat{h} = 1.92 \pm 0.07$. Source data are provided as a Source Data file.

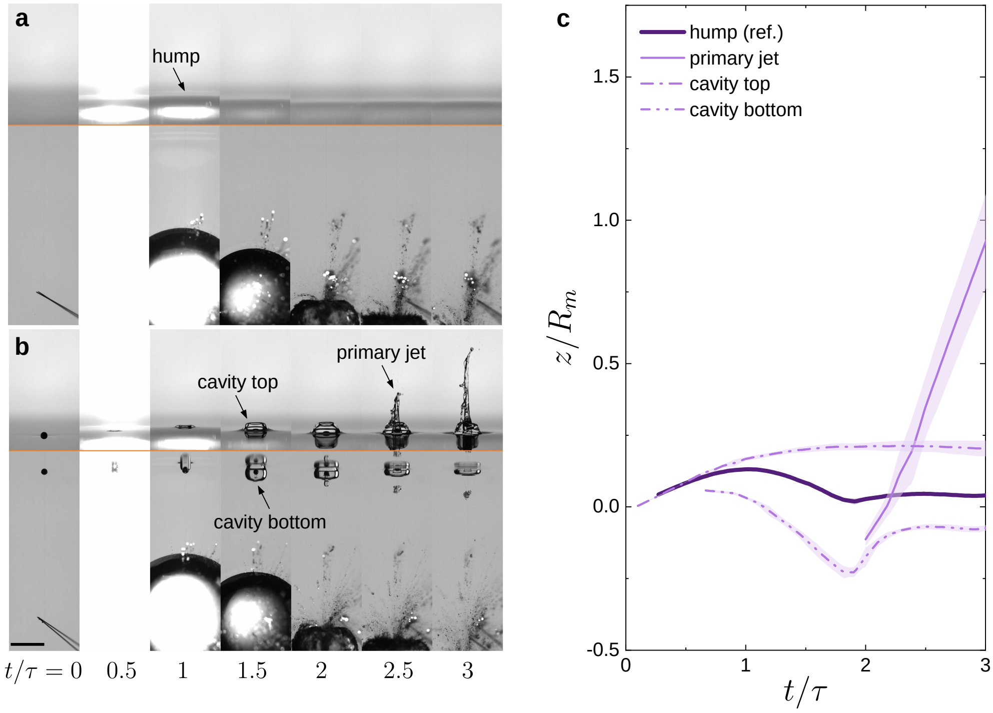

Supplementary Figure 5: **Kinematics of Mode V. a**, Reference case ($\hat{h} = 2.25$). **b**, Snapshots of the Open Cavity Mode ($\hat{h} = 2.25$, $r = 500$ μm, $\rho = 4.4$, $\theta = 111.2°$). Scale bar: 5 mm. **c**, Temporal evolution of featured geometries corresponding to the case shown in **b** and the reference case shown in **a**. The lines are the average results from three experimental runs with $\hat{h} = 2.25 \pm 0.04$. Source data are provided as a Source Data file.

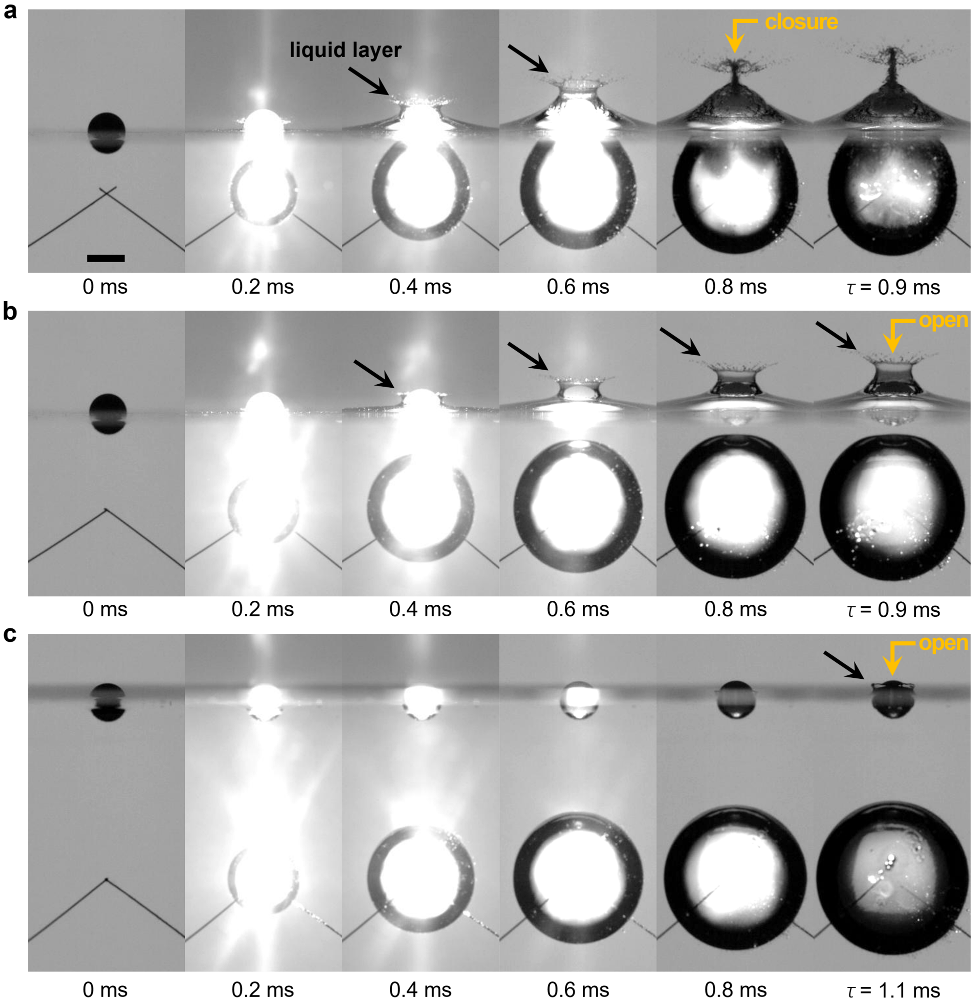

Supplementary Figure 6: **Experimental snapshots illustrate the formation and development of the liquid layer.** In **a**, the liquid layer fully converges above the sphere before $t = \tau$, whereas in **b** and **c**, the layer remains open. The travel distance of the liquid layer remains on the order of the sphere size, fundamentally different from classical water-entry pinch-off depth. Panels **a** and **b** show thinner liquid layers, with splashing filaments and fragmented fine droplets visible at their edges. In contrast, the liquid layer in **c** is not visible until $t = \tau$ and appears thicker with a smooth rim. The dimensionless depths of the spark bubble are $\hat{h} = 0.83$, 1.31, and 2.31 for **a-c**, respectively. Sphere properties: $r = 2500$ μm, $\rho = 1.4$, $\theta = 150.5°$. Scale bar: 5 mm.

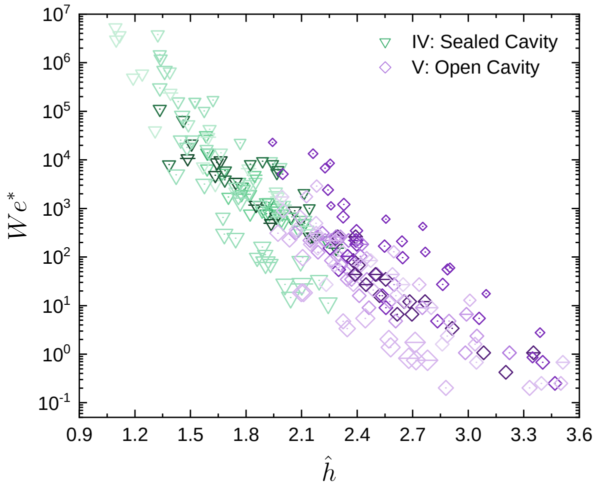

Supplementary Figure 7: **Phase diagram of Modes IV and V plotted against $\hat{h}$ and We^*.** $\hat{h}$ denotes the dimensionless depth of the spark bubble, and We^* is a Weber number defined by the ratio of aerodynamic pressure to Laplace pressure. Source data are provided as a Source Data file.

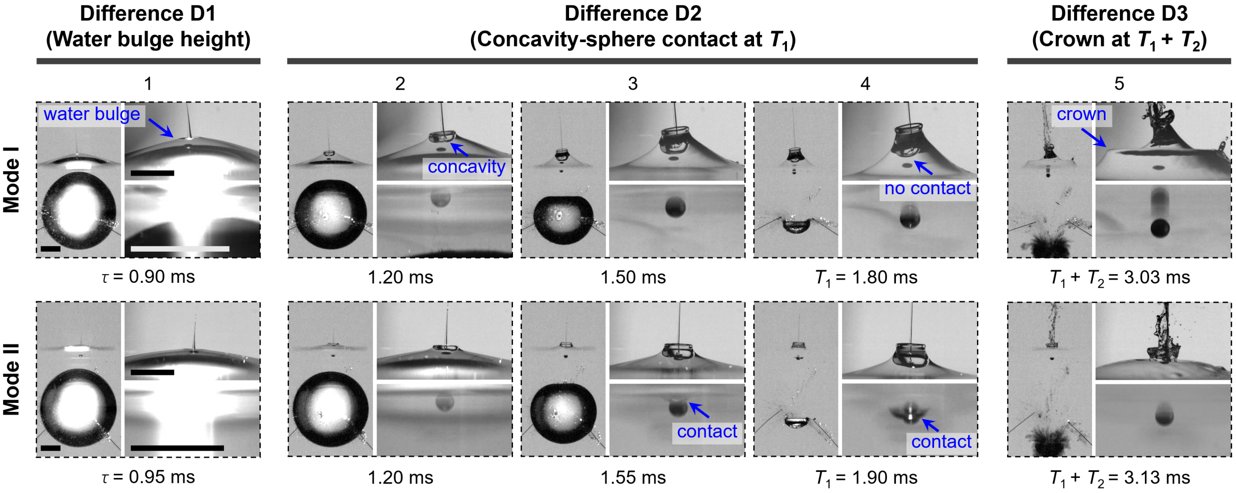

Supplementary Figure 8: **Experimental snapshots illustrating the distinction between Modes I and II.** The figure presents global views, close-ups above the water surface and underwater close-up images. Three observable differences (D1-D3) between the two modes are shown. D1: Height of the water bulge above the initial waterline at $t = \tau$. Mode I (high), Mode II (low); D2: Surface concavity contacts the sphere at $t = T_1$. Mode I (no), Mode II (yes); D3: Crown structure appears at $t = T_1 + T_2$. Mode I (present), Mode II (absent). Among these, D2 is adopted as the sole classification criterion, as it provides a clear binary event directly reflecting the particle-surface interaction. The dimensionless depth of the spark bubble is $\hat{h} = 1.11$ for Mode I and $\hat{h} = 1.35$ for Mode II. Sphere properties: $r = 500$ μm, $\rho = 4.4$, $\theta = 80.8°$. Scale bars: 5 mm.

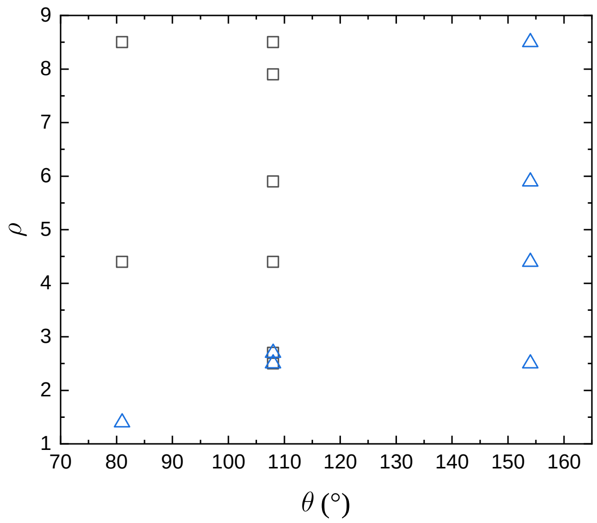

Supplementary Figure 9: Phase diagram showing Modes I (grey squares) and III.a (blue triangles) plotted against the sphere's density ratio ρ and contact angle θ. Source data are provided as a Source Data file.

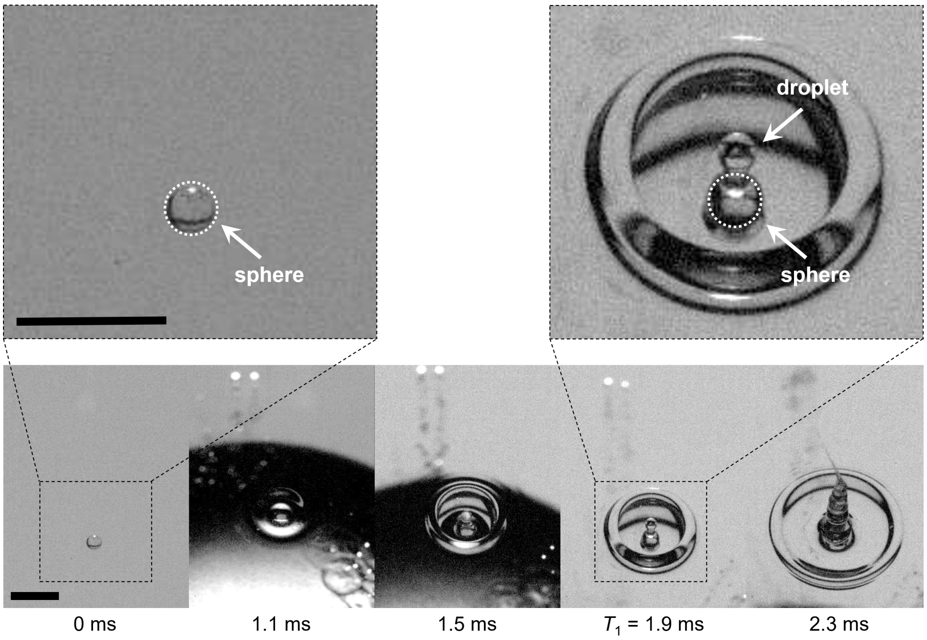

Supplementary Figure 10: **Experimental observation of a micro-scale droplet above the sphere.** At $t = T_1$, a micro-droplet was observed above the sphere, suggesting that the sphere was fully submerged before this time. However, no singular jet was observed before T_1. The interfacial evolution was recorded with a camera positioned at a 60° angle relative to the horizontal plane, providing a top-down view. The dimensionless depth of the spark bubble is $\hat{h} = 1.98$. Sphere properties: $r = 175$ μm, $\rho = 2.5$, $\theta = 105°$. Scale bars: 1 mm.
