# Supplementary material for: Particulate reshapes surface jet dynamics induced by a cavitation bubble
Source: Nat Commun. 2025 Aug 14;16:7562. doi: 10.1038/s41467-025-62936-y (PMC12354815; doi:10.1038/s41467-025-62936-y)
Supplement: Supplementary file 2 — Description of Additional Supplementary Files [file 41467_2025_62936_MOESM2_ESM.pdf]

Description of Additional Supplementary Files

Particulate Reshapes Surface Jet Dynamics Induced by a Cavitation Bubble

Xianggang Cheng[1], Xiao-Peng Chen[1,2*], Zhi-Ming Yuan[3], Laibing Jia[3*]

[1]School of Marine Science and Technology, Northwestern Polytechnical University, Xi'an, 710072, China.
[2]Research & Development Institute of Northwestern Polytechnical University in Shenzhen, Shenzhen, 518057, China.
[3]Department of Naval Architecture, Ocean & Marine Engineering, University of Strathclyde, Glasgow, G4 0LZ, UK.

*Corresponding author(s). E-mail(s): xchen76@nwpu.edu.cn; l.jia@strath.ac.uk;

File Name: Supplementary Movie 1
Description: A movie of temporal evolution for Jet Mode I: Tiered Jet. The experimental parameters are: $\hat{h} = 0.81$, $\rho = 4.4$, and $\theta = 80.8°$. The orange lines separate the views above and below the water surface. A reference case with a flat water surface is displayed on the left panel, with a non-dimensional depth of $\hat{h} = 0.85$ for the spark bubble. The original movies were captured at 7,500 frames per second and played back at 1,000 times slower speed.

File Name: Supplementary Movie 2
Description: A movie of temporal evolution for Jet Mode II: Jet Cavity. The experimental parameters are: $\hat{h} = 1.62$, $\rho = 4.4$, and $\theta = 80.8°$. The orange lines separate the views above and below the water surface. A reference case with a flat water surface is displayed on the left panel, with a non-dimensional depth of $\hat{h} = 1.57$ for the spark bubble. The original movies were captured at 10,000 frames per second and played back at 1,000 times slower speed.

File Name: Supplementary Movie 3
Description: A movie of temporal evolution for Jet Mode III: Cavity Venting. The experimental parameters are: $\hat{h} = 1.00$, $\rho = 1.4$, and $\theta = 82.1°$. The orange lines separate the views above and below the water surface. A reference case with a flat water surface is displayed on the left panel, with a non-dimensional depth of $\hat{h} = 1.02$ for the spark bubble. The original movies were captured at 10,000 frames per second and played back at 1,000 times slower speed.

File Name: Supplementary Movie 4
Description: A movie of temporal evolution for Jet Mode IV: Sealed Cavity. The experimental parameters are: $\hat{h} = 1.93$, $\rho = 4.4$, and $\theta = 111.2°$. The orange lines separate the views above and below the water surface. A reference case with a flat water surface is displayed on the left panel, with a non-dimensional depth of $\hat{h} = 1.95$ for the spark bubble. The original movies were captured at 10,000 frames per second and played back at 1,000 times slower speed.

File Name: Supplementary Movie 5
Description: A movie of temporal evolution for Jet Mode V: Open Cavity. The experimental parameters are: $\hat{h} = 2.25$, $\rho = 4.4$, and $\theta = 111.2°$. The orange lines separate the views above and below the water surface. A reference case with a flat water surface is displayed on the left panel, with a non-dimensional depth of $\hat{h} = 2.25$ for the spark bubble. The original movies were captured at 10,000 frames per second and played back at 1,000 times slower speed.
